# The Effect of Plasma Treated PLGA/MWCNTs-COOH Composite Nanofibers on Nerve Cell Behavior

**DOI:** 10.3390/polym9120713

**Published:** 2017-12-14

**Authors:** Jing Wang, Nuan Chen, Seeram Ramakrishna, Lingling Tian, Xiumei Mo

**Affiliations:** 1 College of Chemistry, Chemical Engineering and Biotechnology, Donghua University, Shanghai 201620, China; wangjing290@126.com; 2 Center for Nanofibers and Nanotechnology, E3-05-14, Department of Mechanical Engineering, Faculty of Engineering, National University of Singapore, 2 Engineering Drive 3, Singapore 117576, Singapore; cnuan@u.nus.edu (N.C.); seeram@nus.edu.sg (S.R.); 3 Guangdong-Hongkong-Macau Institute of CNS Regeneration (GHMICR), Jinan University, Guangzhou 510632, China

**Keywords:** electrospinning, nanofibers, MWCNTs-COOH, air plasma, nerve cells

## Abstract

Electrospun nanofibrous scaffolds which can mimic the architecture of the natural extracellular matrix (ECM) are potential candidates for peripheral nerve repair application. Multi-walled carbon nanotubes (MWCNTs) are used in peripheral nerve repair due to their ability to promote neurite extension and support neural network formation. In this study, surface-modified nanofibrous scaffolds composed of poly(lactic-*co*-glycolic acid) (PLGA) and various ratios of carboxyl-modified MWCNTs (MWCNTs-COOH) (PC0, PC2, PC4 and PC8) were fabricated by electrospinning. The effects of MWCNTs-COOH on the fibers’ morphology, diameter distribution, mechanical properties and surface hydrophilicity were characterized by Scanning Electron Microscopy (SEM), ImageJ software, tensile testing and water contact angle. Furthermore, air plasma treatment was applied to improve the surface hydrophilicity of the scaffolds, and the optimal treatment condition was determined in terms of surface morphology, water contact angle and PC12 cell adhesion. Plasma treated nanofibers (p-PC0, p-PC2, p-PC4 and p-PC8) under optimal treatment conditions were used for further study. PC12 cell proliferation and differentiation were both improved by the addition of MWCNTs-COOH in scaffolds. Additionally, the proliferation and maturation of Schwann cells were enhanced on scaffolds containing MWCNTs-COOH. The neurite outgrowth of rat dorsal root ganglia (DRG) neurons was promoted on MWCNTs-COOH-containing scaffolds, and those cultured on p-PC8 scaffolds showed elongated neurites with a length up to 78.27 μm after 3 days culture. Our results suggested that plasma treated nanofibers under appropriate conditions were able to improve cell attachment. They also demonstrated that plasma treated scaffolds containing MWCNTs-COOH, especially the p-PC8 nanofibrous scaffold could support the proliferation, differentiation, maturation and neurite extension of PC12 cells, Schwann cells and DRG neurons. Therefore, p-PC8 could be a potential candidate for peripheral nerve regeneration application.

## 1. Introduction

Peripheral nerve injuries arising from trauma, cancer, or congenital defects are challenging clinical issues as they affect an increasing number of people, with dramatic consequences such as the loss of sensory or motor function. Approximately 200,000 peripheral nerve repair procedures are performed every year in the United States alone, which causes a huge social burden [1]. Direct end-to-end surgical reconnection is a common method for small injury gaps, while large nerve defects are normally treated with autologous nerve grafts. However, complete axonal reconnection and functional regeneration can rarely be achieved. Thus, tissue engineering scaffolds may serve as an alternative choice for peripheral nerve regeneration.

Electrospun nanofibers have been used extensively as substrates for neural tissue engineering and achieved great success [2,3,4]. Various kinds of biomaterials such as poly(l-lactide acid) (PLLA) [5], poly(caprolactone) (PCL) [6] and poly(lactic-*co*-glycolic acid) (PLGA) [7] have been applied to fabricate nanofibrous scaffolds for nerve regeneration using electrospinning technique. Additionally, conductive biomaterials such as polypyrrole (PPy) [8], polyaniline (PANI) [9] and Poly(3,4-ethylenedioxythiophene) (PEDOT) [10] have also been used for scaffold fabrication for nerve regeneration and to achieve a certain level of conductivity. Carbon nanotubes (CNTs) are another group of conducting materials with unique structural, electrical, and mechanical properties. Multi-walled carbon nanotubes (MWCNTs) are composed of layers of graphite sheets with diameters of up to 100 nm, and this unique structure offers excellent physical and chemical properties which enable wide applications of MWCNTs. Numerous studies have investigated the effect of MWCNTs on neuronal behavior, especially on their ability to promote neurite extension and support neural network formation [11]. Mattson et al. reported the biocompatibility of carbon nanotube for neuronal growth in vitro for the first time, and they demonstrated the ability of MWCNT-layered substrates to support the long-term survival of cultured dissociated hippocampal neurons [12]. Jin et al. fabricated MWCNT-coated electrospun poly(l-lactic acid-*co*-caprolactone) (PLCL) nanofibers and found that the fibers improved neurite outgrowth of both PC12 cells [13] and rat dorsal root ganglia (DRG) neurons [14]. All these results suggest that MWCNTs could be used as a potential scaffold material to improve neural cells response including cell attachment, proliferation and differentiation for neuroregenerative applications.

CNTs were shown to be toxic to cells as a suspension in cell culture media [15]. Most of the reported toxicological data are based on in vitro experiments with certain cell lines. The cytotoxicity can be regulated by functionalization and safety dosage [16]. Modifications can enable CNTs increased water miscibility and reduced toxicity. Electrospinning is one of the most efficient ways to embed CNTs into fabricated composite nanomaterials [17,18]. In this present study, we aimed to fabricate PLGA nanofibers with various proportions of carboxyl modified MWCNTs (MWCNTs-COOH). The effects of MWCNTs-COOH on the properties of composite nanofibers, including morphology, hydrophilic performance, and mechanical properties were evaluated. Since PLGA served as the main component, and knowing the hydrophobic properties of PLGA, it was necessary to improve the surface properties. Plasma treatment is a favorable method to improve surface hydrophilicity and provide better biocompatibility of scaffolds in tissue engineering [19]. Various functional chemical groups, such as the hydroxyl, the carboxyl group and the amino group can be introduced onto the surface of materials by different plasma modifying strategies, and then the surface properties such as wettability, surface roughness and cytocompatibility are changed [20]. In this study, MWCNTs-COOH-containing nanofibers were further treated with air plasma. The change in surface chemistry and hydrophilicity of the scaffolds were characterized by Energy Dispersive X-ray Detector (EDX) and water contact angle, respectively. Furthermore, the influence of plasma treatment on PC12 cells adhesion was investigated. The effect of plasma treated nanofibrous scaffolds with various ratios of MWCNTs-COOH, on the cellular responses of PC12 cells, DRG neurons and Schwann cells, such as cell attachment, proliferation and differentiation were further assessed to investigate the potential of these scaffolds for PNS repair.

## 2. Materials and Methods

### 2.1. Materials

The 75/25 poly(lactic-*co*-glycolic acid) (PLGA) (inherent viscosity 0.75 dL/g) was purchased from Jinan Daigang Biomaterial Co., Ltd., Jinan, China. 1,1,1,3,3,3-Hexafluoro-2-propanol (HFP), glutaraldehyde, Dulbecco’s modified eagle’s medium (DMEM/F12), were purchased from Sigma, St. Louis, MO, USA. Graphitized Multi-Wall Carbon Nanotubes COOH (out diameter: 10–20 nm; length: 1–30 μm; purity: >99.9 wt %; COOH content: 1.0–2.0 wt %) was purchased from Cheaptubes, Cambridgeport, VT, USA. Nerve growth factor (NGF) was purchased from Millipore, Singapore. Rat pheochromocytoma (PC12) cells in the adherent type and rat Schwann cells were obtained from ATCC, Manassas, VA, USA, while fetal bovine serum (FBS), horse serum (HS) and trypsin/EDTA were purchased from GIBCO Invitrogen, Thermo Fisher, Waltham, MA, USA. Alamar Blue (AbD Serotec) was purchased from Chemoscience, Singapore. Schwann cell medium was purchase from Gene-Ethics Asia Pte Ltd., Singapore. Anti-S100 antibody produced in rabbit and anti-NF200 produced in rabbit were purchased from Sigma.

### 2.2. Scaffolds Fabrication

#### 2.2.1. Preparation of the Electrospun Suspensions

PLGA was dissolved into 1,1,1,3,3,3-Hexafluoro-2-propanol (HFP) to form a 25% *w*/*v* solution with magnetic stirring of 300 rpm for 12 h. Then the composite PLGA/MWCNTs-COOH suspensions with different ratios of MWCNTs-COOH were obtained by mixing MWCNTs-COOH powder with PLGA solution with a mass ratio of 2:100, 4:100, 8:100 (MWCNTs-COOH:PLGA). Additionally, 100 μL of span 80 was added to improve the dispersion of MWCNTs-COOH. Subsequently, the suspensions were stirred continuously for 24 h. Finally, the suspensions were ultra-sonicated for 1 h before electrospinning.

#### 2.2.2. Electrospinning

The electrospinning process was the same as that described in previous work [21]. The solution for electrospinning was loaded into a 3 mL syringe with an 18 G blunt stainless needle (diameter 0.84 mm). The high voltage electrostatic generator was supplied by Gamma High Voltage, Ormond Beach, FL, USA. The high voltage for electrospinning was 18 kV, and the flow rate was 1 mL/h. The fabricated nanofibers was collected on a plate covered with aluminum foil, and the distance between the collector and the needle tip was 15 cm. The obtained nanofibrous scaffolds with various proportions of MWCNTs-COOH were dried in a vacuum drying oven (DZF-6050, Liyi, Guangzhou, China) for 24 h and then used for further study. The temperature in the vacuum oven was room temperature. Only the effect of the vacuum was used to further dry the scaffolds. Herein, the labels PC2, PC4 and PC8 correspond to nanofibrous scaffolds with MWCNTs-COOH content of 2, 4 and 8%, respectively. Pure PLGA scaffolds were also fabricated as a control, and labeled as PC0.

#### 2.2.3. Optimization of Plasma Treatment Conditions

In order to further improve the surface hydrophilicity of the fabricated scaffolds, air plasma treatment was conducted for the scaffolds by a plasma cleaner (Model: PDC-001, Harrick Scientific Corporation, Pleasantville, NY, USA). PC0 scaffolds were used to optimize the treatment duration. The radiofrequency power was set as 30 W under vacuum. The effect of air plasma treatment on the scaffold was controlled by changing the processing period. So, plasma discharge was applied to PC0 scaffolds with various durations, including 60, 120, 180 s. The optimized processing period for air plasma treatment was determined by the evaluation results of plasma treated nanofibers by SEM, water contact angle and tensile mechanical properties. Then the scaffolds with MWCNTs-COOH content of 0, 2, 4 and 8% to be used for further study, were all treated with air plasma using the optimized duration, and the obtained samples were labelled as p-PC0, p-PC2, p-PC4 and p-PC8.

### 2.3. Scaffold Characterization

#### 2.3.1. Scanning Electron Microscopy (SEM)

The electrospun nanofibers were gold-coated using sputter coating (JEOL JFC-1200 Fine coater, Tokyo, Japan) and the surface topographies were visualized by SEM (Model S-4300, Hitachi, Tokyo, Japan). The gold-coating process was controlled by the current and period. Here, the current for gold-coating was 10 mA, and the process lasted for 80 s. The diameter distribution of the nanofibers was determined from the SEM images using image analysis software (ImageJ, National Institutes of Health, Bethesda, MD, USA). 100 nanofibers were chosen from the SEM images for the diameter measurement.

#### 2.3.2. Transmission Electron Microscopy (TEM)

The distribution of MWCNTs-COOH in the fibers was observed using transmission electronic microscopy (JEM-3010, JEOL, Tokyo, Japan) at 200 kV. The samples for TEM observation were prepared by collecting the fibers onto a carbon film-coated copper grid directly for about 8 s. Then the samples were dried in a vacuum drying oven for 24 h to remove residual solvent completely.

#### 2.3.3. Water Contact Angle

Hydrophilicity of the nanofibers with various ratios of MWCNTs-COOH and pure PLGA nanofibers with different plasma treatment duration was measured by water contact angle using VCA Optima Surface Analysis System (AST products, Billerica, MA, USA). The detailed steps were the same as those described in previous work [22]. Firstly, the nanofiber mat was placed on the testing plate and kept smooth. Subsequently, 0.05 mL of distilled water was dropped slowly onto the surface of the nanofiber mat. The images of the water drop on the nanofiber mat were recorded by a camera software in the testing system after the droplet was stable. Then, the water contact angle was measured with the measuring tool in the testing system. Three different points of each sample were measured, and the average value was calculated.

#### 2.3.4. Surface Chemistry Analysis

The surface chemistry of pure PLGA nanofibers before and after plasma treatment was analyzed by energy-dispersive X-ray (EDX). The changes in three kinds of elements including carbon, oxygen and nitrogen on the fiber surface were evaluated with EDX. The nanofibers were visualized under SEM without any melt coating. EDX analysis was done by Quantax 70 (Bruker, Berlin, Germany) mounted on a SEM.

#### 2.3.5. Mechanical Properties

The mechanical properties of the nanofibers with various ratios of MWCNTs-COOH and pure PLGA nanofibers with various plasma treatment duration were measured with stress-strain analysis performed using a universal materials tester (H5K-S, Hounsfield, Salfords, UK) at room temperature. The samples were cut into rectangular specimens with dimensions of 30 mm × 10 mm. Then, the thickness was measured with a digital micrometer (Digimatic Micrometer Series 293 MDC-MX Lite, Mitutoyo, Kawasaki, Japan). Subsequently, two sides of the rectangular specimen were fixed with the two clamps of the tester. After that, mechanical testing was conducted with a stretch speed of 10 mm/min. The stretch was stopped until the specimen was completely broken. The reported data of tensile strength, elongation, Young’s Modulus and toughness represent the average results of six tests.

### 2.4. PC12 Cell Culture In Vitro

PC12 cells were cultured using the same method as described in previous work [1]. Briefly, PC12 cells were cultured in growth medium which was composed of 84% DMEM/F12, 10% HS, 5% FBS, and 1% antibiotic/antimycotic solution in a 75 cm^2^ cell culture flask. Cells were incubated in a CO_2_ Incubator (MCO-19 AIC (UV), Panasonic Healthcare, Gunma, Tokyo, Japan) at 37 °C and 5% CO_2_. The culture medium was changed once every 2 days. After the cells were grown to confluency, they were detached with trypsin/EDTA and seeded on different nanofibrous scaffolds with a density of 20,000 cells per well for proliferation and differentiation tests and 30,000 cells per well for the adhesion test.

### 2.5. The Adhesion of PC12 Cells on PC0 Nanofibers with Different Plasma Treatment Duration

Plasma treatment was applied to improve the hydrophilicity and biocompatibility of the electrospun nanofibers. In order to determine the optimized treatment duration, adhesion of PC12 cells on pure PLGA nanofibers with various plasma treatment duration was evaluated. The PC12 cells used here were adherent type. Cell adhesion is the first and most critical step that occurs after seeding the cells on the scaffolds, followed by cell proliferation and differentiation. The adhesion of PC12 cells was evaluated by counting the number of cells attached on the scaffold, while the cell number was measured with Alamar blue assay as specified by the manufacturer. The cells were allowed to be attached to the scaffold for 12 h after cell seeding. Then, the medium was removed and the scaffold with cells was rinsed thrice with PBS to remove the unattached cells. The number of cells attached on the scaffold was further evaluated with Alamar blue. In detail, 800 μL Alamar blue solution (10% Alamar blue, 90% DMEM/F12 medium; *v*/*v*) was added to each well followed by incubation at 37 °C for 4 h. Then the Alamar blue solution was pipetted into 96-well plate and the fluorescence at 560 nm (excitation)/590 nm (emission) was read by a Varioskan flash reader (Thermo Fisher Scientific, Waltham, MA, USA). The number of cells was positively proportional to the fluorescence intensity.

### 2.6. The Proliferation of PC12 and Schwann Cells

The optimized plasma treatment time was determined based on the above test results. In order to further evaluate the influence of nanofibers with various content of MWCNTs-COOH on nerve cell behavior, all samples for the following research were treated with air plasma for an optimized duration.

Cell proliferation of both PC12 and Schwann cells were evaluated with Alamar blue assay as described above. The cells were seeded on p-PC0, p-PC2, p-PC4, p-PC8 nanofibrous scaffolds which were sterilized for 5 h under UV light in a biological safety cabinet (Bioclassic Class II Series Type A2, Gelman Laminar Airflow Systems, Singapore). The UV lamp for sterilization came with the biological safety cabinet. The UV length was 250–260 nm. After seeding on the scaffolds, the cell culture plate was put into the CO_2_ incubator (MCO-19AIC (UV), Panasonic Healthcare, Gunma, Japan) at 37 °C and 5% CO_2_. The culture medium was changed once every 2 days. The cell proliferation was tested after 1, 4 and 7 days of culture in vitro.

### 2.7. The Neuronal Differentiation of PC12 Cells

For the differentiation study of PC12 cells cultured on p-PC0, p-PC2, p-PC4, p-PC8 nanofibrous scaffolds, the medium used was changed to a differential medium composed of DMEM/F12 supplemented with 1% HS, 0.5% FBS, 1% antibiotic/antimitotic solution and 100 ng mL^−1^ NGF 1 day after cell seeding.

#### 2.7.1. Morphology of Differentiated PC12 Cells

SEM was applied to evaluate the morphology of differentiated PC12 cells cultured on different scaffolds. Firstly, the scaffolds with cells were washed twice with PBS and fixed with 10% formalin for 20 min at room temperature (about 25 °C). The PBS containing 137 mM sodium chloride, 2.7 mM potassium chloride and 10 mM phosphate buffer used here was pH 7.4 ± 0.2. After that, the scaffold-cell constructs were rinsed 3 times with 1 mL distilled water, and dehydrated through a series of graded ethanol (10, 70, 90, and 100%). Finally, the samples were air-dried for 12 h, sputter-coated with gold, and observed by SEM.

#### 2.7.2. Immunostaining of Neurofilament 200 (NF200)

The neural differentiation of PC12 cells was further evaluated by immunofluorescence staining of a nerve specific protein-NF200. After 7 days of cell culture in differentiation medium on different scaffolds, the cell-scaffolds constructs were rinsed thrice with PBS and then fixed with 10% formalin for 20 min. After that, the samples were permeabilized with 0.1% Triton X-100 for 5 min followed by incubation with 3% *w*/*v* BSA aqueous solution for 90 min to block the nonspecific binding. Subsequently, the sample were stained with anti-NF200 produced in rabbit as the primary antibody at dilution of 1:100 (Sigma) for 2 h at room temperature. The anti-Rabbit IgG (whole molecule)–FITC antibody was diluted 200 times and used as the secondary antibody to combine with the primary antibody for 1 h. The immunostained samples were mounted onto glass slides with mounting medium with DAPI and observed under laser scanning confocal microscope (Zeiss LSM700, Oberkochen, Germany).

### 2.8. Neurite Outgrowth of Dorsal Root Ganglia (DRG) Neurons

To assess the neurite outgrowth of DRG neurons, rat DRG neurons were seeded on different scaffolds at a density of 10,000 cells per well in a 24-well cell culture plate, and cultured in DMEM/F12 with 10% FBS and 50 ng mL^−1^ NGF in a 37 °C, 5% (*v*/*v*) CO_2_ incubator for 5 days. For immunofluorescence staining of specific protein-NF160, the cells were fixed with 10% formalin for 20 min, treated with 0.1% Triton X-100 for 5 min, and blocked with 3% *w*/*v* BSA aqueous solution for 90 min. Then the samples were immunostained with the primary antibody solution overnight at 4 °C, followed by incubation with Goat anti-Rabbit IgG Alexa 488 (Invitrogen, Camarillo, CA, USA) diluted 1:200 in PBS as the secondary antibody for 1 h. After staining with DAPI for 15 min, the outgrowth of immunostained DRG neurons was visualized under LSCM.

### 2.9. Morphologies of Schwann Cells

The morphological analysis of Schwann cells on p-PC0, p-PC2, p-PC4, p-PC8 nanofibrous scaffolds was evaluated with immunofluorescence staining for S100 protein, a commonly used astrocyte marker. Cells cultured on different scaffolds were fixed with 10% formalin at day 5 followed by permeabilization with 0.1% Trixton-X100 for 5 min. Then, the nonspecific binding was blocked with 3% *w*/*v* BSA aqueous solution for 90 min. After that, the samples were incubated with rabbit anti-S100 antibody (dilution 1:100; Sigma) at 4 °C overnight. Subsequently, samples were stained with FITC conjugated secondary antibody (dilution 1:100; Sigma) at room temperature for 1 h, and nuclei was stained with DAPI for 15 min. Finally, the samples were mounted onto glass slides and visualized under a laser scanning confocal microscope (LSCM, Zeiss LSM700).

### 2.10. Statistical Analysis

Statistical analysis was performed using ANOVA and Student’s *t*-test. All data were expressed as mean ± standard deviation (SD). In all statistical evaluations, *p* < 0.05 was considered statistically significant.

## 3. Results and Discussion

**Figure 1:**
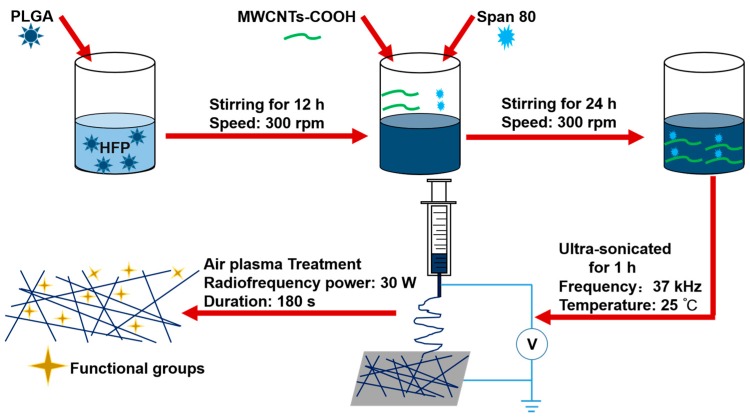
Schematic illustration of solution preparation, electrospinning and air plasma treatment.

During this study, the PLGA/MWCNTs-COOH composite nanofibers were fabricated via electrospinning and the nanofibers were treated with air plasma to improve their surface hydrophilicity. The process of fabrication is schematically demonstrated in Figure 1.

### 3.1. Characterization of Electrospun Nanofibers

**Figure 2:**
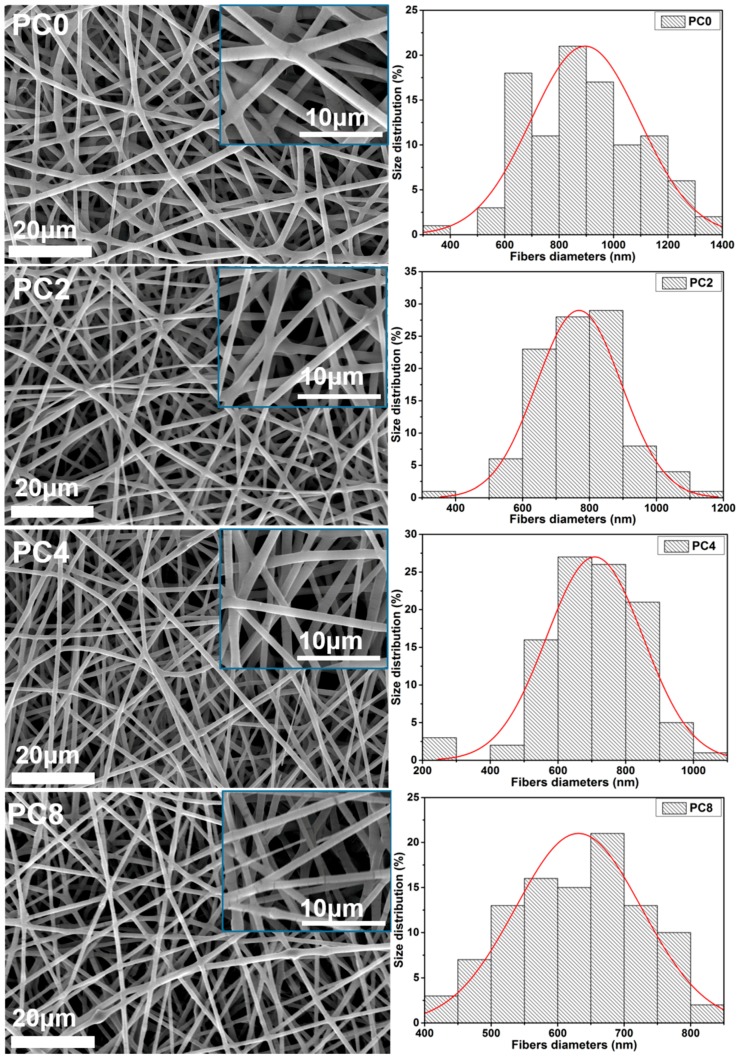
SEM images of electrospun nanofibers as well as the distribution of their diameters.

Figure 2 shows the SEM images of PC0, PC2, PC4 and PC8 nanofibers, along with their diameter distributions. All the nanofibers were smooth in appearance without bead-like structure, which indicated that the MWCNTs-COOH dispersed uniformly in PLGA fibers without serious aggregation. The fiber diameter decreased with the addition of MWCNTs-COOH into polymer matrix, and the average diameter also decreased gradually with the increase of MWCNTs-COOH content due to the increase in electric conductivity. The fiber diameter dropped from 913.35 ± 185.19 nm to 631.41 ± 94.74 nm with the addition of 8% MWCNTs-COOH (as shown in Table 1).

**Figure 3:**
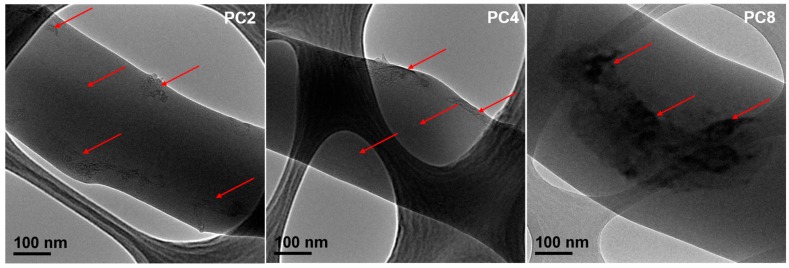
Selected TEM images of PC2, PC4 and PC8 electrospun nanofibers.

The distribution of MWCNTs-COOH was evaluated by TEM. As images displayed in Figure 3 show, the MWCNTs-COOH could be distinguished in the fibers. In many regions of the composite nanofibers the embedded MWCNTs-COOH appeared to be well-oriented along the fiber axis. However, in some regions, MWCNTs-COOH protruded out of the composite fibers making the composite fibers appear rough. Some MWCNTs-COOH in fibers, especially in the PC8 scaffold, exhibited some degree of aggregation and formed bundles in the polymer matrix. Therefore, the content of MWCNTs-COOH played an important role in influencing their distribution in nanofibers. This was mainly influenced by the dispersion of original MWCNTs-COOH in the electrospinning suspensions. With the increasing ratio of MWCNTs-COOH in electrospinning suspensions, it became more difficult to form homogeneous dispersion.

**Figure 4:**
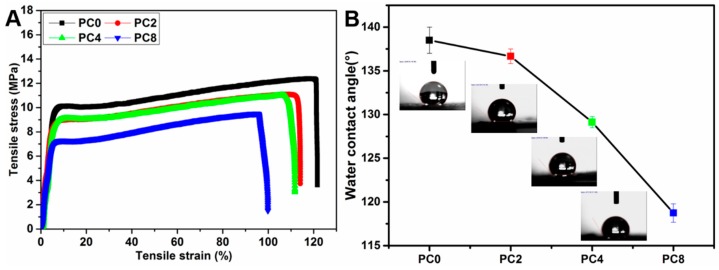
(**A**) The stress-strain curves and (**B**) water contact angles of PC0, PC2, PC4 and PC8 nanofibrous scaffolds.

The stress-strain curves of PLGA nanofibers with various content of MWCNTs-COOH are presented in Figure 4A. The mechanical properties, such as Young’s modulus, maximum tensile strength and elongation at break for various scaffolds are shown in Table 1. The Young’s modulus, maximum tensile strength and elongation at break all demonstrate an obvious decrease with the MWCNTs-COOH content increasing from 0 to 8% in the PLGA matrix. The dispersion of MWCNTs-COOH in the polymer matrix was the most important factor in determining the mechanical properties of the PLGA/MWCNTs-COOH composite nanofibers. MWCNTs were well aligned along the nanofiber axis when the content of the MWCNTs was within certain limits [23], and the mechanical properties were enhanced. However, when the proportion further increased and went beyond that range, the mechanical properties declined due to the maldistribution of MWCNTs in the polymer matrix [24]. Agglomeration of the nanotubes occurred with the further increase in MWCNTs concentration, which subsequently decreased their degree of orientation in respect to the fiber axis and weakened the interaction between nanotubes and polymer molecular chains. As a result, the polymer chains could not transfer the load to the MWCNTs, resulting in decreasing tensile strength. Therefore, in our study the decrease in mechanical properties with the incorporation of MWCNTs-COOH may be due to the random dispersion of MWCNTs in the PLGA matrix as a result of the relatively large quantity of MWCNTs [24,25].

The water contact angles of PC0, PC2, PC4 and PC8 nanofibers were shown in Table 1 and Figure 4B. The water contact angle decreased from 138.5 ± 1.5° to 118.7 ± 1.05° when the MWCNTs-COOH content increased from 0 to 8%, suggesting that the hydrophilicity of the nanofibers was improved in the presence of MWCNTs-COOH. The existence of MWCNTs-COOH on the surface of the fiber can reduce the water contact angle to some extent due to the hydrophilic carboxyl groups. As we know, the hydrophilicity of the scaffolds is very important since it can influence the initial cell adhesion. Although the addition of MWCNTs-COOH was able to improve the surface hydrophilicity of the scaffolds to a certain extent, the effect was insufficient because hydrophobic PLGA was the main component of the scaffolds. In order to further improve the hydrophilicity of the scaffolds, plasma treatment was applied to introduce functional groups on the surface.

### 3.2. Optimization of Plasma Treatment Condition

**Figure 5:**
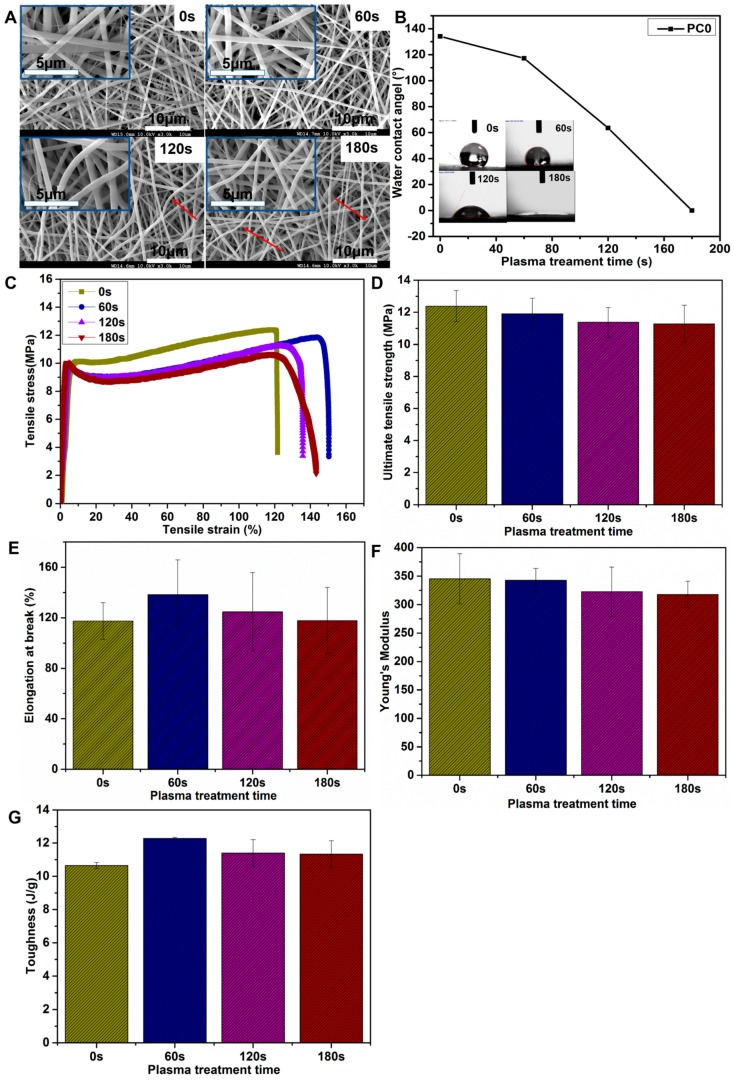
(**A**) SEM images and (**B**) water contact angles of PC0 nanofibers after a plasma treatment time of 0, 60, 120, and 180 s; (**C**) The tensile stress-strain curves of PC0 nanofibers with various plasma treatment duration, and (**D**) calculated ultimate tensile strength, (**E**) elongation at break, (**F**) Young’s modulus and (**G**) toughness.

In this study, air plasma was used for PC0 nanofibers with various processing periods to optimize the treatment duration. The scaffolds were evaluated by SEM, water contact angle, tensile test and EDX after the plasma treatment. From the SEM images shown in Figure 5A, it was found that the morphology of fibers did not show significant changes with a 60 s treatment duration, while treatment with 120 and 180 s caused a slight curl in the fibers (indicated by red arrows in Figure 5A). The water contact angle of PC0 nanofibers with different plasma treatment times is shown in Figure 5B. We found that the water contact angle decreased from 134.1° to 0°, which indicated that the surface hydrophilicity of the scaffolds was improved significantly within the appropriate air plasma treatment time. The tensile stress-strain curves of PC0 nanofibers with various plasma treatment times are shown in Figure 5C, and the calculated ultimate tensile strength, elongation at break and Young’s modulus are presented in Figure 5D–F. There was no statistically significant differences in all three aspects of ultimate tensile strength, elongation at break and Young’s modulus between any two groups, which indicated that the mechanical properties of PC0 nanofibers appeared not to have been affected within 180 s plasma treatment duration. In order to further investigate the flexibility of scaffolds with different plasma treatment time, the toughness of the scaffold was calculated according to [26]. As shown in Figure 5G, the toughness of the scaffold slightly increased after plasma treatment, but there was no statistical difference between groups. The results indicated that the flexibility of the scaffold may be improved with enough plasma treatment, and the indistinctive changes in our study maybe due to the short processing time.

**Figure 6:**
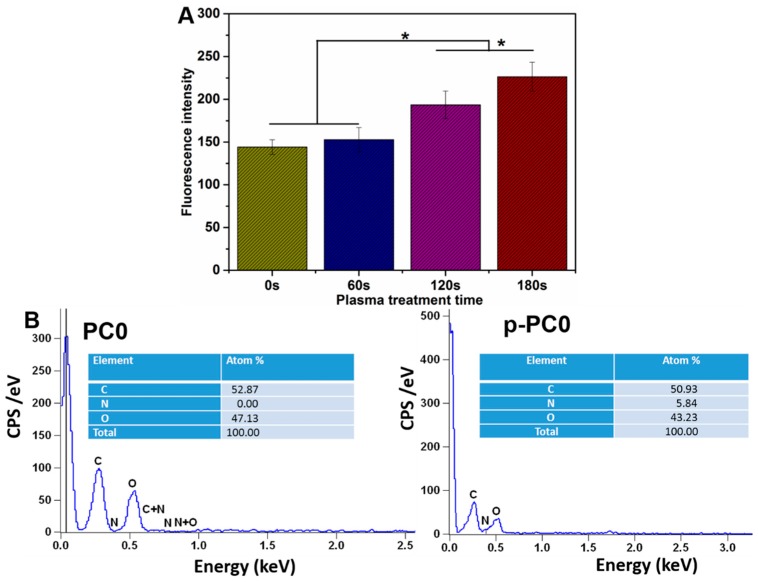
(**A**) The adhesion of PC12 cells on PC0 nanofibers with different plasma treatment times; (**B**) EDX spectra and atomic ratios (%) of PC0 nanofibers before and after plasma treatment of 180 s.

In order to further confirm the optimum duration, the adhesion of PC12 cells on PC0 scaffolds with different plasma treatment time was assessed after 12 h of cell culture. As shown in Figure 6A, we found that the nanofibers with 120 and 180 s plasma treatment duration were able to provide more hydrophilic and adhesive surface for cell attachment, which was in accordance with water contact angle results. EDX was used to evaluate the changes of surface chemistry of scaffolds after plasma treatment, and Figure 6B shows the results of PC0 nanofibers with 0 and 180 s plasma treatment duration. There was no nitrogen observed on the surface of PC0 nanofibers before plasma treatment, while the ratio of nitrogen increased from 0 to 5.84% after being treated for 180 s with air plasma. This result demonstrated that a functional group, such as amino or another nitrogenous group, was introduced on the surface of PC0 nanofibers with air plasma treatment. Therefore, the optimized duration of plasma treatment was chosen as 180 s based on the above results, and scaffolds used for following studies were all treated with air plasma for 180 s.

### 3.3. Proliferation Study of PC12 and Schwann Cells

**Figure 7:**
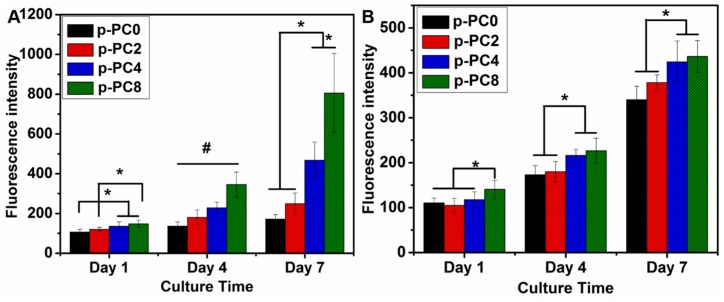
The proliferation of PC12 (**A**) and Schwann cells (**B**) on p-PC0, p-PC2, p-PC4, and p-PC8 nanofibers. (* = significant difference between the labelled two groups; # = significant difference between any two groups. *p* < 0.05).

In order to further evaluate the effect of scaffolds with various proportions of MWCNTs-COOH on cell response, PC0, PC2, PC4, PC8 nanofibers with air plasma treatment for 180 s, which were labelled as p-PC0, p-PC2, p-PC4, and p-PC8 were used for the proliferation study of both PC12 and Schwann cells. As shown in Figure 7A, PC12 cells proliferated gradually on all scaffolds from day 1 to day 7. However, the cell proliferation showed differences on different scaffolds. On day 1, cells cultured on p-PC4 and p-PC8 nanofibers had better growth than those cultured on p-PC0, p-PC2 scaffolds. After 4 days of cell culture, we observed that PC12 cells on all nanofibers containing MWCNTs-COOH (p-PC2, p-PC4 and p-PC8) grew better when compared with those on PLGA nanofibers without MWCNTs-COOH (p-PC0), and the cells proliferated faster as the MWCNTs-COOH content increased. By the 7th day, the numbers of cells on p-PC4 and p-PC8 nanofibers were significantly higher than those on p-PC0 and p-PC2 nanofibers, and cell numbers were highest on p-PC8. For all three time points, p-PC8 nanofibers resulted in better PC12 cells proliferation compared to all the others. The proliferation of Schwann cells is shown in Figure 7B. There was no obvious difference between all scaffolds except for p-PC8 nanofibers, which had the highest number of live cells after 1 day of culture. On both day 4 and day 7, the cell numbers on p-PC4 and p-PC8 nanofibers surpassed those on both p-PC0 and p-PC2 nanofibers. Additionally, there was no significant difference between p-PC4 and p-PC8, as well as p-PC0 and p-PC2 nanofibers. All of these results suggest that the scaffolds containing 8% MWCNTs-COOH can greatly improve the proliferation and growth of both PC12 and Schwann cells, which may also contribute to favorable protein adsorption from the culture medium as a result of the presence of MWCNTs-COOH.

### 3.4. Neuronal Differentiation Study of PC12 Cells

#### 3.4.1. Morphology Observation by SEM

**Figure 8:**
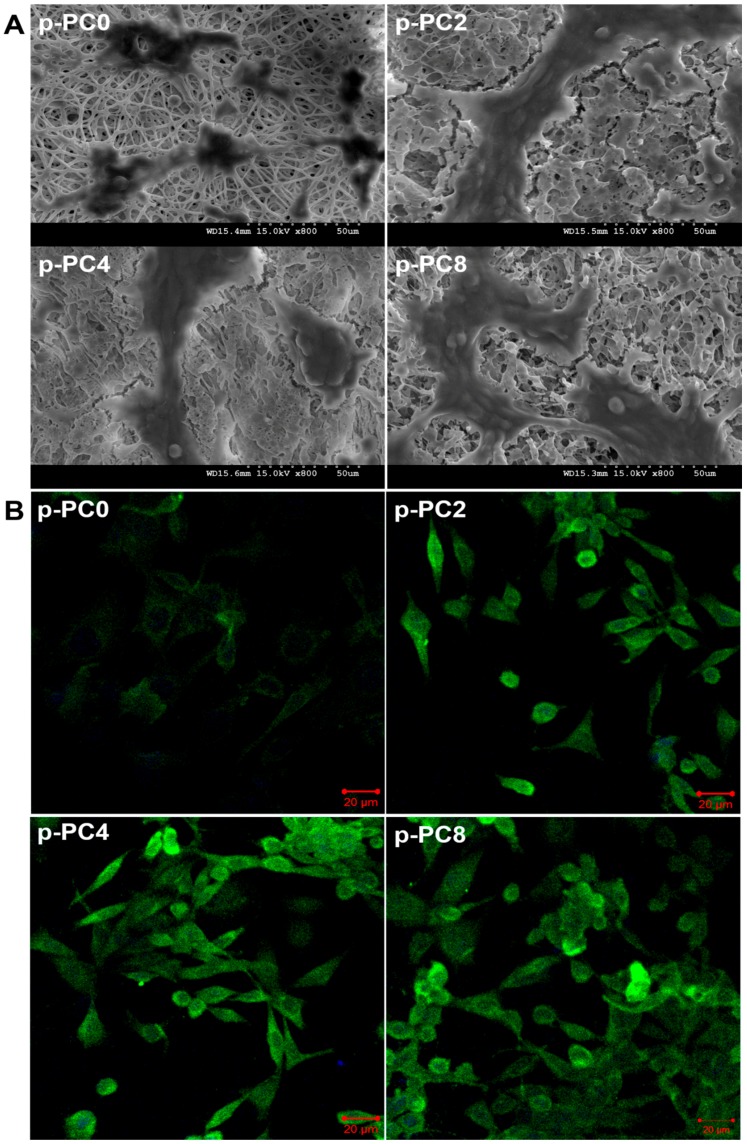
(**A**) SEM images of differentiated PC12 cells and (**B**) NF200 expression of PC12 cells in differentiation medium on p-PC0, p-PC2, p-PC4 and p-PC8 nanofibers.

The morphology of the differentiated PC12 cells grown on different scaffolds were observed using SEM (Figure 8A). As we know, the undifferentiated PC12 cells were small and sphere-shaped. After being treated with NGF containing differentiated medium and culturing for 7 days, a few PC12 cells grown on p-PC0 nanofibers showed an elongated shape, while there were more PC12 cell projected neurites on MWCNTs-COOH-containing scaffolds, which confirmed the differentiation of PC12 cells. Furthermore, on scaffolds containing MWCNTs-COOH, the cells tended to gather together. These results suggested that the MWCNTs-COOH in the scaffold may contribute to the neuronal differentiation of PC12 cells and cell–cell contact.

#### 3.4.2. Immunostaining of NF200

Neurofilament proteins are synthesized in the neuronal perikarya and assembled to form filaments which are slowly transported within the axons [1]. The expression of neuronal protein (NF200) was evaluated to further confirm the differentiation of PC12 cells on different scaffolds. The immunofluorescence staining of NF200 could also compare the cell phenotype and neurite outgrowth on different scaffolds. As shown in Figure 8B, the cells cultured on all scaffolds expressed NF200 protein, but the scaffolds containing MWCNTs-COOH showed more support for NF200 expression compared to pure PLGA scaffolds (p-PC0). Under the stimulation of NGF, PC12 cells enabled differentiation into neuron-like appearance with extended neurites. PC12 cells grown on scaffolds containing MWCNTs-COOH showed more neurites formed compared to those on pure PLGA scaffolds (p-PC0). This result was consistent with the SEM observations. All the results indicated that the differentiation of PC12 cells was improved by the MWCNTs-COOH in scaffolds in a certain range.

**Figure 9:**
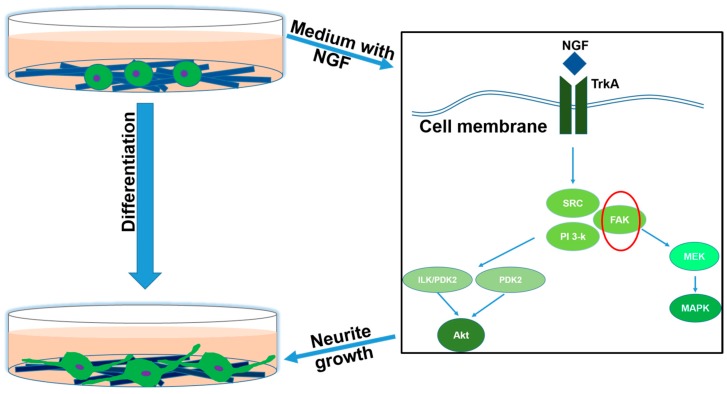
The schematic illustration of NGF induced differentiation and neurite growth of PC12 cells in this study. The combination of NGF and TrkA resulted in activation of Src and FAK, which resulted in the sustained activation of the downstream signaling. Activation of Akt downstream of PI 3-K is essential for neurite growth in this system. Additionally, signaling via MEK/MAPK may also leads to axonal extension. However, it is dispensable for neurite growth of NGF-responsive neurons.

The differentiation and neurite growth of PC12 cells induced by NGF was schematically illustrated in Figure 9. A dramatic change in phenotype of PC12 occurred while they responded to NGF. Previous studies have found that both Src and FAK are the key regulators of NGF and integrin-dependent signaling in adult NGF-responsive neurons [27]. Activation of TrkA by NGF resulted in activation of Src and FAK, which resulted in the sustained activation of the downstream signaling intermediate Akt. Previous studies found that the expression of FAK was up-regulated by MWCNTs [14], so the mechanism of MWCNTs-COOH-containing scaffolds for the promotion of the differentiation of PC12 cells was mainly by up-regulating FAK expression. In addition, the influx of Ca ions and associated plasma membrane recycling could also influence the neurite outgrowth [28]. Hence, the enhanced neurite growth by scaffolds containing MWCNTs-COOH may also be attributed to the changes in the intracellular Ca^2+^ level. However, the complete mechanism of this needs further investigation.

### 3.5. Neurite Length of Rat DRG Neurons on Nanofibers

**Figure 10:**
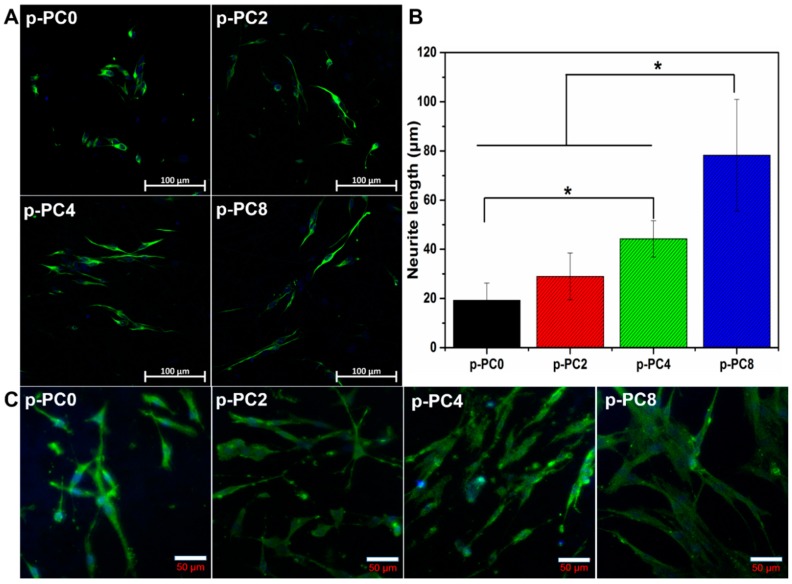
(**A**) LSCM images of rat DRG neurons on different nanofibers after 3 days of culture; (**B**) Neurite length of rat DRG neurons measured with ImageJ software; (**C**) LSCM images of Schwann cells cultured on different nanofibers after 7 days of culture. Green: S100 antibody; Blue: nuclei. Data was mean ± deviation; *n* =10; * = significant difference at *p* < 0.05.

To further evaluate the influence of nanofibers containing MWCNTs-COOH on the neurite extension of DRG neurons, rat DRG neurons cultured on different nanofibers were stained with NF160 protein and observed under microscope (Figure 10A). Additionally, the neurite length of DRG neurons was measured using ImageJ software, and the results are presented in Figure 10B. The average length of DRG neurons on p-PC0, p-PC2, p-PC4 and p-PC8 nanofibers after culturing for 3 days were 19.23 ± 7.03, 28.96 ± 9.53, 44.25 ± 7.38 and 78.27 ± 22.70 μm, respectively. Comparison of the mean neurite lengths of the DRG neurons cultured on different nanofibers revealed that the neurites on the MWCNTs-COOH-containing scaffolds, particularly p-PC4 and p-PC8 nanofibers, were significantly longer. The p-PC8 nanofibers were the ones that promoted the neurite extension most. MWCNTs-COOH in nanofibrous scaffolds demonstrated positive effects on the neurite growth of DRG neurons. The mechanism of MWCNTs-COOH in the promotion of neurite extension of DRG neurons may be the same as that of PC12 cells, and a more in-depth investigation is necessary.

### 3.6. The Morphology and Phenotype of Schwann Cells on Nanofibers

Schwann cells are the main glial cells in the peripheral nerve system, which play a crucial role in supporting neurite growth by producing neurotrophic factors. The results above showed the promotion of Schwann cells proliferation on scaffolds containing MWCNTs-COOH. To further confirm the interaction of Schwann cells with MWCNTs-COOH-containing nanofibers, the morphology and phenotype of Schwann cells were evaluated by immunostaining of S100 antibody and then visualized under a confocal microscope. LSCM images in Figure 10C show that there were cells that appeared rounded, exhibiting an immature morphology, on the p-PC0 scaffold while on scaffolds containing MWCNTs-COOH, almost all cells exhibited a typical mature stage appearance with a spindle-like shape In particular, the cells showed longer bio-polar extension on the p-PC8 scaffold,. The matured myelinating Schwann cells are able to up-regulate many myelin gene expressions and release various neurotrophins such as NGF [28], which is able to attract injured neurons and aid axon elongation. Therefore, the MWCNTs-COOH in the scaffold may enhance the maturity of Schwann cells.

## 4. Conclusions

The enhancement of nerve cell behaviors, such as adhesion, proliferation, differentiation and maturation, should be taken into account in the design of peripheral nerve tissue engineered scaffolds. In this study, we prepared PLGA nanofibrous scaffolds containing various proportions of MWCNTs-COOH via electrospinning, and air plasma treatment was applied to improve the surface hydrophilicity of the scaffolds. Plasma treated scaffolds were more favorable to PC12 cells adhesion. The addition of MWCNTs-COOH in scaffolds was beneficial to PC12 cell proliferation, neuronal differentiation, and Schwann cell maturity. The p-PC8 nanofibrous scaffold provided a superior substrate for neural differentiation of PC12 cells and neurite extension of rat DRG neurons. Collectively, p-PC8 nanofibers could be a promising tissue engineered scaffold for peripheral nerve regeneration.

## Figures and Tables

**Table 1:** Average diameters, mechanical properties and water contact angles of scaffolds containing different MWCNTs-COOH content.

Scaffolds	Average diameter (nm)	Maximum tensile strength (MPa)	Elongation at break (%)	Young’s modulus (MPa)	Water contact angle (°)
PC0	913.35 ± 185.19	12.39 ± 0.70	117.46 ± 6.55	348.85 ± 49.85	138.50 ± 1.50
PC2	768.55 ± 127.20 *	11.15 ± 1.01	96.36 ± 14.86	328.34 ± 20.53	136.66 ± 0.84
PC4	708.81 ± 142.45 *	11.13 ± 0.89 *	99.68 ± 20.02	304.58 ± 13.23	129.13 ± 0.64 *
PC8	631.41 ± 94.74 *	9.12 ± 0.69 *	81.84 ± 14.20 *	245.63 ± 19.59 *	118.73 ± 1.05 *

* Indicates significant difference in comparison with PC0, *p* < 0.05.
